# Preoperative semen quality is superior to the quality shortly after orchiectomy in patients with testicular germ cell tumour – a retrospective study from two centres in Germany

**DOI:** 10.1186/s12610-025-00252-7

**Published:** 2025-02-18

**Authors:** Klaus-Peter Dieckmann, Johanna Hochmuth-Tisch, Andrea Salzbrunn, Cord Matthies, Kathrein von Kopylow, Christian Wülfing, Uwe Pichlmeier, Armin Soave, Christian Guido Ruf

**Affiliations:** 1 Asklepios Klinik Altona, Urologische Abteilung, Hamburg, Germany; 2 Westküstenklinikum Heide, Frauenklinik, Heide, Germany; 3 Klinik und Poliklinik für Dermatologie und Venerologie, Bereich Andrologie, Universitätsklinikum Hamburg-Eppendorf, Hamburg, Germany; 4 Abteilung Urologie, Bundeswehrkrankenhaus Hamburg, Hamburg, Germany; 5 Institute of Medical Biometry and Epidemiology, Universitätsklinikum Hamburg-Eppendorf, Hamburg, Germany; 6 Urologische Klinik und Poliklinik, Universitätsklinikum Hamburg-Eppendorf, Hamburg, Germany; 7 Bundeswehrkrankenhaus Ulm, Department of Urology, Oberer Eselsberg 40, Ulm, 89081 Germany

**Keywords:** Testicular germ cell tumour, Semen quality, Sperm cryopreservation, Sperm count, Sperm motility, Seminoma, Nonseminoma

## Abstract

**Background:**

Sperm cryopreservation in patients with testicular germ cell tumours (GCTs) is traditionally performed after orchiectomy. But, some evidence suggests preoperative semen quality to be superior. We aimed to clarify the optimal time-point of cryopreservation.In a retrospective study, semen quality of 163 patients analysed preoperatively was compared with 242 patients analysed shortly after orchiectomy. Descriptive statistical methods with standard tests for comparisons were employed along with stratified analyses regarding the influence of clinical factors.

**Results:**

All major semen parameters were significantly better in the preoperative group: median ejaculate volume (3 ml preoperatively vs. 2 ml postoperatively); median total sperm count (56.9 x10^6^vs.13 x 10^6^), median progressive motility (40% vs. 25%); azoospermia (4.9% vs. 14.9%). Stratified analysis of subgroups did not reveal significant impact of particular clinical factors on the superiority of preoperative semen quality. Limitations relate to the design of group comparison rather than intraindividual longitudinal comparisons and to selective inclusion of patients opting for cryopreservation.

**Conclusions:**

In GCT patients, semen quality before orchiectomy is significantly superior to that found immediately after surgery. This superiority encompasses all major semen quality parameters. Of particular note is a threefold increase of azoospermia postoperatively. GCT patients are best advised to have cryopreservation performed before orchiectomy.

## Résumé

**Contexte:**

La cryoconservation des spermatozoïdes chez les patients atteints de tumeurs germinales testiculaires est traditionnellement réalisée après une orchidectomie. Mais certaines données suggèrent que la qualité du sperme préopératoire est supérieure. Notre objectif était de clarifier le moment optimal de la cryoconservation. Dans une étude rétrospective, la qualité du sperme a été analysée en préopératoire chez 163 patients et a été comparée à celle du sperme analysé chez 242 patients peu de temps après l’orchidectomie. Des méthodes statistiques descriptives avec tests standards pour les comparaisons ont été utilisées, ainsi que des analyses stratifiées concernant l’influence des facteurs cliniques.

**Résultats:**

Tous les principaux paramètres du sperme étaient significativement meilleurs dans le groupe préopératoire : volume médian de l’éjaculat (3 ml avant l’opération versus 2 ml après l’opération) ; nombre médian total de spermatozoïdes (57millions versus 13 millions par éjaculat), motilité progressive médiane (40 % contre 25 %), azoospermie (4,9 % contre 14,9 %). L’analyse stratifiée des sous-groupes n’a pas révélé d’impact significatif de facteurs cliniques particuliers sur la supériorité de la qualité du sperme préopératoire. Les limites de l’étude sont liées à la conception de la comparaison de groupe plutôt qu’aux comparaisons longitudinales intra-individuelles, ainsi qu’à l’inclusion sélective de patients optant pour la cryoconservation.

**Conclusions:**

Chez les patients atteints de tumeurs germinales testiculaires, la qualité du sperme avant orchidectomie est nettement supérieure à celle constatée immédiatement après la chirurgie. Cette supériorité englobe tous les principaux paramètres de qualité du sperme. Il convient de noter en particulier que l’azoospermie a triplé en postopératoire. Il est préférable que les patients atteints de tumeurs germinales testiculaires réalisent une cryoconservation avant l’orchidectomie.

**Mots clés:**

Tumeur germinale testiculaire ; Qualité du Sperme ; Cryoconservation du Sperme ; Spermatozoïdes; Motilité des Spermatozoïdes ; Séminome ; non-séminome

## Background

Testicular germ cell tumours (GCTs) can effectively be cured in the vast majority of patients [1]. However, clinical management involves a variety of significant long-term sequelae [2]. Impaired fertility secondary to cisplatin-based chemotherapy is a paramount issue since testicular GCTs afflict patients in their third and fourth decade of life when family planning is usually not yet completed [3]. Moreover, testicular GCT is a disease damaging the generative tissue, and hence, sperm production in GCT patients is generally poorer than in healthy men even before treatment [4–6]. Accordingly, guide-lines recommend sperm cryopreservation prior to any gonadotoxic treatment [7, 8]. Less clear is the particular time-point at which cryopreservation should be performed. Traditionally, sperm banking was conducted after orchiectomy before the start of any further treatment [9–11]. The rationale for this scheduling was the view that valuable time could be lost by conducting cryopreservation prior to surgery thereby compromising the chance of cure. However, recent experience has challenged the traditional timing of sperm banking for two reasons: First, testicular GCT is a very well curable malignancy nowadays, and orchiectomy though inevitable, is clearly not an urgent step in the management of the disease. Delay of surgery of around two or three weeks does not negatively impact cure rates [12–15]. Second, recent investigations reported sperm quality to be better in preoperative semen samples compared to postoperative measurements [16]. However, timing of cryopreservation remained an issue of debate, since several studies found no differences between pre- and postoperative semen quality [17] and some even reported superior quality after orchiectomy [18]. The goal of the present study was to contribute comprehensive information for counselling GCT-patients with respect to the optimal time-point of sperm cryopreservation.

## Methods

### Patients

Semen analysis was performed in consecutive patients with histologically proven testicular GCT treated in Bundeswehrkrankenhaus Hamburg (BWKH) during 2012 – 2023 and in Asklepios Klinik Altona (AKA) during 2016–2023, all of whom requested sperm cryopreservation prior to systemic treatment. Relevant patient data were retrospectively abstracted from electronic hospital archives. Other features of this series had been reported previously [6].

The patients were stratified according to the time point of semen collection. In the preoperative group, semen analysis was performed before orchiectomy. The postoperative group was analysed after orchiectomy but before the start of additional therapy with an interval from surgery to sperm donation of less than three weeks in the vast majority of cases.

The following clinical and oncological characteristics were registered in each patient: age (years), histology (seminoma or nonseminoma), clinical stage (CS) according to UICC (CS1, CS2a,b; CS2c; CS3), tumour size according to the pathohistological report (cm); serum levels of alpha fetoprotein (AFP) and human chorionic gonadotropin (bHCG) in relation to the upper limit of norm (ULN).

The Ethical Committee of Ärztekammer Hamburg approved the study (2021-100629-BO-ff). Written consent on participation in the study was obtained from all patients. All study activities were in accordance with the World Medical Association´s Declaration of Helsinki as adopted at the 64th General Assembly in October 2013.

### Semen analysis

All semen samples were processed according to the World Health Organization (WHO) criteria [19]. The majority of participants kept a minimum three-day interval of sexual abstinence before the examination with no considerable difference between the two cohorts regarding abstinence time. Semen analyses of patients of BWKH were performed in that institution. AKA patients had their analyses done in the Department of Andrology, University Medical Centre Hamburg-Eppendorf. For each participant, the following semen parameters were registered: ejaculate volume (EV; ml); total sperm count (TSC; n × 10^6^), progressive motility (%), and total motility (%). A diagnosis of azoospermia (TSC 0 × 10^6^) was usually based on a single examination because time constraints regarding cancer treatment precluded further examinations. Sperm morphology was not assessed.

### Statistical analysis

Clinical data and semen measurement results of each participant were originally filed in a standard database (MS Excel, version 2019). After thorough validation of entries, the data base was transferred to SAS software package version 9.4 (SAS Institute, Cary, NC, USA) for final statistical analysis.

The preoperative and postoperative group were compared to each other with regard to the following parameters: (1) ejaculate volume; (2) proportion of < 1.4 ml ejaculate volume; (3) TSC; (4) proportion of TSC < 39 × 10^6^; (5) proportion of azoospermia; (6) proportion of progressive motility; (7) proportion of total motility; (8) proportion of progressive motility < 30%. To evaluate possible interrelationships between the various parameters, the frequencies of three joint factors combining the presumably most relevant single parameters with threshold values according to the latest WHO Manual edition were calculated and tabulated: (9) progressive motility < 30% & TSC < 39 × 10^6^; (10) progressive motility < 30% & TSC ≥ 39 × 10^6^; (11) progressive motility ≥ 30% & TSC < 39 × 10^6^.

To look for any interactions of clinical factors with semen results, stratified analyses of semen parameters were performed in subgroups defined by the clinical features. Accordingly, preoperative and postoperative semen parameters were compared to each other in 5 subgroups with stratifications as follows: age (< 30 years vs. ≥ 30 years); histology (seminoma vs. nonseminoma); clinical stage (CS1 vs. > CS1); AFP (≤ ULN vs. > ULN); bHCG (≤ ULN vs. > ULN). In this subanalysis, we included only ejaculate volume, TSC, progressive motility and the joint parameter progressive motility < 30% & TSC < 39 × 10^6^.

For the description of location and dispersion of quantitative variables, medians, first quartiles (Q1) and third quartiles (Q3) were calculated. For binary variables, percentages and exact 95% Clopper Pearson confidence intervals (CIs) were derived. Chi square tests were applied for comparisons of the two main cohorts with respect to binary variables, replaced by Fisher´s exact test in case of expected cell counts less than 5. For comparison of quantitative variables, Wilcoxon two sample tests with t approximation were used.

In order to investigate whether the pattern of difference between pre- and postoperative data is heterogeneously distributed between subgroups defined by clinical factors, analyses of variance (for quantitative variables) and logistic regression analyses (for binary variables) were calculated including not only the respective clinical variable and the time-factor in the model but also the associated interaction term. Due to the skewness of the distribution, a log transformation was first necessary to “normalize” the distributions of EV and TSC. All statistical tests were two-sided. Statistical significance was assumed at *p* < 0.05.

## Results

A total of 405 patients were included, 163 (40.7%) and 242 (59.3%) in the preoperative and postoperative group, respectively. Clinical details of the two populations are listed in Table 1. The groups are consistent with regard to tumour size and frequencies of marker level elevations (all p > 0.05). However, the postoperative group consisted of significantly more younger patients than the preoperative group (p = 0.0067) and included significantly more nonseminoma cases (*p* < 0.0001), and more advanced clinical stages (*p* < 0.0001).

**Table 1:** Clinical characteristics in the two patient populations

time-point of semen analysis	before orchiectomy	after orchiectomy
Age *		
eligible (n)	163	242
< 30 years (n; %)	71 (43.6%)	144 (59.5%)
30–39 years (n; %)	81 (49.7%)	85 (35.1%)
> 40 years (n; %)	11 (6.8%)	13 (5.4%)
Histology *		
eligible (n)	163	241
Seminoma (n; %)	92 (56.4%)	81 (33.6%)
Nonseminoma (n; %)	71 (43.5%)	160 (66.4%)
Clinical stage *		
eligible (n)	158	237
CS1 (n; %)	128 (81.0%)	130 (54.8%)
CS2a,b (n; %)	24 (15.2%)	68 (28.7%)
CS2c (n; %)	3 (1.9%)	9 (3.8%)
CS3 (n; %)	3 (1.9%)	30 (12.7%)
**Tumour size**		
eligible (n)	156	191
< 2 cm (n; %)	38 (24.4%)	36 (18.8%)
2.1–4 cm (n; %)	83 (53.2%)	105 (55.0%)
> 4 cm (n; %)	35 (22.4%)	50 (26.2%)
**AFP- serum level**		
eligible (n)	159	196
< ULN	125 (78.6%)	131 (66.8%)
1—10 × ULN	19 (11.9%)	37 (18.9%)
11—50 × ULN	12 (7.6%)	18 (9.2%)
> 50 × ULN	3 (1.9%)	10 (5.1%)
**beta HCG- serum level**		
eligible (n)	159	198
< ULN	89 (55.9%)	122 (61.6%)
1—10 × ULN	38 (23.9%)	34 (17.2%)
11—50 × ULN	16 (10.1%)	18 (9.1%)
> 50 × ULN	16 (10.1%)	24 (12.1%)

The data show some disparities among the two patient populations. The postoperative group consisted of significantly more cases of younger age, more nonseminoma cases, and more patients with advanced clinical stages (marked with asterisk) than the preoperative population. Chi square test was used for statistical comparisons. CS clinical stage; ULN upper limit of norm, AFP alpha fetoprotein, bHCG human beta chorionic gonadotropin

Comparison of the two main groups revealed significantly superior semen quality in the preoperative group with regard to all major semen parameters (all details in Table 2). The most striking difference was found with respect to the median total sperm count which was 56.9 × 10^6^ in the preoperative group but only 13 × 10^6^ in the postoperative group (*p* < 0.0001). Likewise, the rate of azoospermia was 4.9% and 14.9% in the preoperative and postoperative group, respectively. Also, the joint parameter progressive motility < 30% & TSC < 39 × 10^6^ indicating over-all poor semen quality was significantly more frequently observed in postoperative cases (51.1% vs 28.7%).

**Table 2:** Comparison of semen quality parameters betwen preoperative and postoperative group

		before orchiectomy	after orchiectomy	p-value
**ejaculate volume (ml)**	n	163	242	
	median	3	2	
	Q1; Q3	2; 4.2	2; 3	< 0.0001
**proportion EV < 1.4 ml (%)**	n	163	242	
	%	6.1%	8.3%	
	95% CI	3.0%; 11.0%	5.1%; 12.5%	0.4223
**total sperm count (n)**	n	163	242	
	median	56.9	13	
	Q1; Q3	13.7; 126.6	2; 48	< 0.0001
**TSC, proportion < 39 × 10** ^**6**^	n	163	242	
	%	41.7%	70.7%	
	95% CI	34.1%; 49.7%	64.6%; 76.3%	< 0.0001
**proportion azoospermia**	n	163	242	
	%	4.9%	14.9%	
	95% CI	2.1%; 9.4%	10.6%; 20.0%	0.0016
**progressive motility (%)**	n	*n* = 157	*n* = 221	
	median	40	25	
	Q1; Q3	25; 51.3	0.3; 36.5	< 0.0001
**proportion motility < 30%**	n	*n* = 157	*n* = 221	
	%	36.9%	67.0%	
	95% CI	29.4%; 45.0%	60.3%; 73.1%	< 0.0001
**motility < 30% & TSC < 39 × 10** ^**6**^	n	157	221	
	%	28.7%	51.1%	
	95% CI	21.7%; 36.4%	44.3%; 57.9%	< 0.0001
**motility < 30% & TSC ≥ 39 × 10** ^**6**^	n	157	221	
	%	8.3%	15.8%	
	95% CI	4.5%; 13.7%	11.3%; 21.3%	0.0297
**motility ≥ 30% & TSC < 39 × 10** ^**6**^	n	157	221	
	%	12.7%	20.8%	
	95% CI	8.0%; 19.0%	15.7%; 26.8%	0.0415

This table shows the main results of the study: all semen parameters are significantly more favourable in the preoperative group, with the exception of proportion of ejaculate volume <1.4 ml. Statistical comparisons were made with the chi square test for binary variables and with the Wilcoxon two sample test for quantitative variables. EV ejaculate volume; TSC total sperm count; CI confidence interval; Q1 first quartile, Q3 third quartile

Regarding the possible influence of clinical factors, the clinical subgroup analyses uniformly revealed superior semen parameters in preoperative cases with no significant difference between stratifications (all details in Table 3).

**Table 3:** Comparison of preoperative with postoperative semen parameters within particular clinical categories

		**ejaculate volume (ml)**					**total sperm count (n × 10** ^**6**^ **)**					**progressive motility (%)**					**motility < 30% & TSC < 39 × 10** ^**6**^				
		**preoperative**		**postoperative**			**preoperative**		**postoperative**			**preoperative**		**postoperative**			**preoperative**		**postoperative**		
		(n)	median (Q1; Q3)	(n)	median (Q1; Q3)	p	(n)	median (Q1; Q3)	(n)	median (Q1; Q3)	p	(n)	median (Q1; Q3)	(n)	median (Q1; Q3)	p	(n)	(%)	(n)	(%)	p
**age**	< 30 yrs	(71)	**3.1** (2.1; 4.1)	(144)	**2.0** (2.0; 3.0)	< 0.0001	(71)	**70.4** (23; 128.8)	(144)	**9.5** (2.3; 40)	< 0.0001	(69)	**40** (25; 55.5)	(129)	**25** (5; 40)	< 0.0001	(69)	**24.6**	(129)	**46.5**	0.0026
	≥ 30 yrs	(92)	**3.0** (2.0; 4.4)	(98)	**2.2** (2.0; 3.0)	0.0045	(92)	**39.7** (6.7; 125.2)	(98)	**20** (2; 55)	0.0016	(88)	**40** (25; 49.8)	(92)	**14.4** (0; 25)	< 0.0001	(88)	**31.8**	(92)	**57.6**	0.0005
	test for age*time interaction					0.3203					0.1177					0.3474					0.8418
**histology**	seminoma	(92)	**3.0** (2.1; 4)	(81)	**2.0** (2.0; 3.0)	0.0002	(92)	**41.9** (6; 126.5)	(81)	**10.0** (2.0; 36.0)	< 0.0001	(89)	**39.5** (25; 49)	(77)	**12.5** (0; 25)	< 0.0001	(89)	**34.8**	(77)	**62.3**	0.0004
	nonseminoma	(71)	**3.0** (2.0; 4.4)	(160)	**2** (2; 3)	0.0002	(71)	**60** (24; 131.3)	(160)	**13.8** (3.0; 50.3)	< 0.0001	(68)	**40** (25; 58.3)	(143)	**25** (5; 40)	< 0.0001	(68)	**20.6**	(143)	**44.8**	0.0007
	test for histology*time interaction					0.7843					0.5295					0.3697					0.9848
**clinical stage**	CS1	(128)	**3.0** (2.0; 4.3)	(130)	**2.5** (2.0; 3.0)	0.0002	(128)	**56.2** (12.5; 133.2)	(130)	**15.0** (2.5; 48.0)	< 0.0001	(123)	**40** (25; 51.5)	(123)	**25** (4; 40)	< 0.0001	(123)	**29.3**	(123)	**44.7**	0.0121
	> CS1	(30)	**3.0** (2.0; 4.0)	(107)	**2.0** (2.0; 3.0)	0.0047	(30)	**59.9** (21.0; 105.3)	(107)	**10.0** (2.0; 40.0)	< 0.0001	(29)	**40** (25.0; 48.5)	(94)	**20.5** (0; 25)	< 0.0001	(29)	**20.7**	(94)	**76.4**	0.0002
	test for CS*time interaction					0.9824					0.1885					0.1700					0.0531
**AFP**	≤ ULN	(125)	**3.0** (2.1; 4.0)	(131)	**2.0** (2.0; 3.0)	< 0.0001	(125)	**45.0** (12.0; 128.7)	(131)	**10** (2; 45)	< 0.0001	(121)	**40** (25; 50)	(124)	**25** (0; 35)	< 0.0001	(121)	**30.6**	(124)	**51.6**	0.0008
	> ULN	(34)	**3.5** (2.0; 4.5)	(65)	**2.5** (2.0; 3.0)	0.0507	(34)	**72.5** (23.0; 119.2)	(65)	**14** (3; 50)	0.0004	(33)	**40** (25; 56)	(55)	**25** (5; 37.5)	0.0006	(33)	**18.2**	(55)	**56.4**	0.0004
	test for AFP*time interaction					0.3158					0.9610					0.7839					0.1382
**bHCG**	≤ ULN	(89)	**3.0** (2.1; 4.6)	(122)	**2.5** (2.0; 3.0)	< 0.0001	(89)	**40.0** (7.5; 126.6)	(122)	**14.0** (2.5; 48.0)	< 0.0001	(87)	**40** (25; 49.5)	(114)	**25** (0.3; 33)	< 0.0001	(87)	**29.9**	(114)	**52.6**	0.0012
	> ULN	(70)	**3.0** (2.0; 4.0)	(76)	**2.0** (2.0; 3.0)	0.0032	(70)	**66.4** (21.0; 128.4)	(76)	**7.8** (2.0; 48.0)	< 0.0001	(67)	**40** (25; 58)	(67)	**25** (0; 40)	< 0.0001	(67)	**25.4**	(67)	**52.2**	0.0014
	test for bHCG*time interaction					0.2024					0.4344					0.8174					0.6601

This analysis shows that the clinical disparities between the two populations (age, histology, CS) are probably not involving an important effect regarding the overall result of better semen quality at preoperative measurements. All subcategories of patients age, histology, clinical stages and tumour marker elevation levels, respectively, uniformly reveal superior semen quality parameters in the preoperative setting. Moreover, the extents of difference (pre- versus postoperative measurement)) between the subgroups (test for time interaction) are not significantly different. Analysis of variance was performed for log (EV), log (TSC) and motility. For binary variables a logistic model was applied to evaluate any potential interaction. CS clinical stage, TSC total sperm count, ULN upper limit of norm, Q1 first quartile, Q3 third quartile, AFP alpha fetoprotein, bHCG beta human chorionic gonadotropin

## Discussion

The crucial result of the present study is the substantial superiority of pre-orchiectomy semen quality over the quality shortly after surgery in GCT patients and this advantage applies to all relevant semen quality parameters.

The most striking difference between preoperative and postoperative semen samples relates to the total sperm count which is more than fourfold higher in preoperative samples than in postoperatives (56.9 × 10^6^ vs. 13 × 10^6^). Accordingly, the azoospermia rate increased from 4.9% before surgery to 14.9% postoperatively. Sperm motility is likewise inferior in postoperative cases highlighted by the proportion of progressive motility < 30% which almost doubled from 36.9% (preoperatively) to 67.0%, postoperatively. Also, the joint parameter progressive motility < 30% &TSC < 39 × 10^6^ featuring the two most important adverse facets of semen quality is almost twice as frequent in postoperative cases as in preoperative patients (51.1% vs. 28.7%).

Superior semen quality at the time before orchiectomy was already noted by Weissbach in 1978 [20]. In 1999, a Danish study [21] reported decreased sperm concentration after orchiectomy in 30 of 35 cases while motility did not deteriorate. A report from Italy also examining intraindividual longitudinal changes in 30 patients, confirmed the significant postoperative decrease of sperm counts and also noted that the decrease was largest in nonseminomas [22]. A UK study found both significantly decreased sperm counts and motility postoperatively in 40 GCT patients examined longitudinally [23]. Further support came from a large study from France [16] that evaluated 155 GCT patients both pre- and postoperatively. A significant reduction of total sperm count was found but no changes regarding sperm motility. The same authors confirmed this finding by comparing a cohort of patients examined preoperatively (*n* = 320) with another cohort examined postoperatively (*n* = 674). Our results are consistent with the four studies regarding sperm counts [16, 21–23]. However, motility data are inconsistent among the studies. Both, the Danish and French study observed no change of motility postoperatively, while impaired motility in the postoperative setting is found in the UK study and this investigation. The underlying reasons for the variance of motility data among the studies remain elusive.

The superiority of preoperative semen quality is not undisputed. At least seven studies reported equal semen quality before and after orchiectomy [9, 17, 18, 24–27], one even noted a trend towards better sperm quality postoperatively [18]. But, caution is advised since all studies encompass small patient numbers. Statistical chance effects may be involved in these studies, particularly, since semen quality parameters are subject to large variability among patients [23]. Methodologically, comparison of preoperative with postoperative semen samples was mostly not conducted in a systematic way, and the finding of equal semen quality before and after surgery was an explorative finding in most of these works. The formal evidence from these studies is certainly modest.

In the present study, the patients had not been randomly assigned to either group. Therefore, the clinical factors governing the timing of semen analysis need to be considered. The patients of the postoperative group were significantly younger, had a higher proportion of nonseminomas and significantly more advanced clinical stages than patients of the preoperative subgroup. It could thus be speculated that the younger age and advanced clinical stages had led the care-givers of these patients to expedited treatment without preoperative sperm banking. Other putative factors for relinquishing preoperative cryopreservation such as lack of counselling or deficits of specific experience appear improbable, since all patients of the present study were treated in two testicular cancer units with high expertise in the management of this disease. Financial aspects cannot entirely be ruled out since the federal law for covering the costs of sperm cryopreservation came into practice only late during the observation period of the present investigation [28].

To analyse if the divergent clinical characteristics might have contributed to the differences between the semen results of the two groups, separate analyses of semen quality in five subgroups were performed with stratifications for the clinical factors. These investigations confirmed the marked superiority of preoperative semen quality in all of these subgroups with no differences between subgroup-specific stratifications. Thus, the clinical heterogeneity among the two groups did not significantly translate into differences of semen quality between the groups.

The superiority of preoperative semen quality over the quality found immediately after surgery appears to be a robust result because firstly, the numerical differences in the semen parameters between the two groups are quite distinct, secondly, all sperm parameters evaluated showed homologous results, and thirdly, the stratified subanalyses of clinical factors revealed no substantial impact of these elements. Moreover, our result is consistent with four well-designed previous studies [16, 21–23].

The biological reason for the poorer semen quality after orchiectomy is probably the loss of germinal tissue through surgery [16]. Histological studies of tumour-bearing testes documented full spermatogenesis in the seminiferous tubules adjacent to the GCT in 40—67% of the cases [29–33]. Accordingly, ONCO-TESE, the surgical extraction of vital spermatozoa from tissue of tumour-afflicted testes had successfully been used for artificial conception [34–37]. The retrieval of vital spermatozoa from the vas deferens and epididymis of tumour-afflicted testes had also been documented [38]. Taken together, there is abundant evidence for active spermatogenesis even in cancerous testes. Orchiectomy thus involves a substantial loss of active sperm generating tissue. Consequently, ejaculates obtained postoperatively must be expected to contain lower sperm counts than preoperative samples. The biological reasons for reduced ejaculate volume after surgery remain unresolved since ejaculate volume is only minimally influenced by testicular function. Nonetheless, the finding of larger ejaculate volumes before orchiectomy is clinically relevant, since it underscores the recommendation of sperm cryopreservation before surgery.

In the present study, 59.3% of the consecutive patients performed their cryopreservation after orchiectomy. This frequency accords with the current pattern of care in Europe [11, 16, 26, 39]. However, our data clearly demonstrate much better semen quality at the time before orchiectomy [16, 21–23]. There is much of evidence for spontaneous recovery of sperm quality in a large proportion of patients after 2 – 5 years [40, 41]. However, not all will recover and the time-interval until recovery may vary depending on the kind of treatment applied after orchiectomy [42–44].

Furthermore, patients confronted with the diagnosis of testicular cancer are subject to time constraints and therefore, waiting for spontaneous recovery of semen quality after orchiectomy is clearly not advisable for the majority of patients. Although expedited treatment is usually not required except for the far advanced GCT cases, cancer-directed treatment should be instituted with no great delay after orchiectomy to ensure optimal oncological outcome. In real life, the next steps of treatment are not clear at the time of orchiectomy in most of the GCT patients. Definitive clinical staging will become evident only some days after orchiectomy, mainly because postoperative tumour marker levels are required for clinical decision-making. Thus, timely sperm banking is probably beneficial, particularly in view of the undecided further management at first diagnosis. Given the chances of improvement of sperm parameters over time an expectant strategy with re-analysis of semen quality some months after surgery could be an option particularly for those without need for additional gonadotoxic treatment and those without urgent need for utilizing their sperm for assisted conception.

Although modern technologies of assisted reproduction such as intracytoplasmatic sperm injection (ICSI) can be successful in the presence of only few viable sperms, cryopreservation should be offered to all GCT patients preferably at the time where sperm counts are highest, which is the time before orchiectomy. Preoperative rather than postoperative cryopreservation is particularly substantiated by the high prevalence of patients with extremely poor semen quality shortly after orchiectomy. The median total sperm count is almost 4 times lower after orchiectomy (13 versus 56.9 [× 10^6^]).

Nearly 15% of patients had even azoospermia postoperatively opposed to only 4.9% preoperatively. Attempts of cryopreservation would be futile in these individuals. Another factor with likely negative impact on ICSI outcomes is very low motility, and noteworthy, the frequency of very low motility (< 30%) is almost twofold in postoperative cases (67%) compared to the preoperative cohort (36.9%). The over-all low semen quality after orchiectomy is best featured by the joint parameter progressive motility < 30% &TSC < 39 × 10^6^ demonstrating the two most important adverse aspects of semen quality in as many as 51% of patients after orchiectomy while these aspects are present in only 28.7% before surgery.

A theoretical argument against cryopreservation before orchiectomy could be the concern that spermatozoa originating from the cancer afflicted testicle could be negatively affected by cancer-associated metabolic processes and might therefore be less robust and might even involve the risk of untoward traits for the newborn, particularly, if in vitro conception is performed with such sperms [45]. However, only sparse data are available to corroborate this concern. In fact, a recent review on outcome of ICSI procedures with cryopreserved sperms from GCT patients did not reveal increased rates of congenital malformations [46]. Yet, it must be acknowledged that the review had included both preoperative and postoperative sperm banking. More importantly, in a review of outcomes of 21 ICSI- procedures after sperm retrieval from cancer-bearing testes (ONCO-TESE), 18 pregnancies were registered with 15 live births and 3 miscarriages [47]. In aggregate, the risk of inherited harm from utilizing preorchiectomy semen samples for ICSI in GCT patients does probably not exceed the natural rate of congenital anomalies and this theoretical concern does clearly not justify the exclusion of preoperative sperm banking.

### Limitations of the study

A major weakness of this retrospective study is certainly the use of group comparisons of semen quality instead of intra-individual longitudinal comparisons. Possibly, the latter study design would be most informative. However, patients would have to produce two semen samples perioperatively. The loss of one testicle undoubtedly represents a substantial emotional burden for the patient with potential consequences on sexuality [48]. Therefore, it may be expected that a considerable number of patients would not comply with a repeat examination, rendering longitudinal studies likewise susceptible to selection bias. Confounding of the study results by disparities between the preoperative and postoperative group regarding age, histology and clinical stages cannot entirely be ruled out although the stratified subgroup analyses did not disclose such effects (Table 3). In the present study, only patients opting for cryopreservation were considered but not those forgoing sperm banking. As no more than one third of all GCT patients in Germany opt for cryopreservation [28] a certain selection bias cannot be ruled out. Minor weaknesses of the study relate to the facts that semen examinations were performed in two different laboratories and that the accrual times of the patients differed slightly among the two participating institutions. Sperm morphology was not assessed in this study due to its unavailability in a number of cases and it was deemed dispensable for the aim of the study, since several previous studies did not include morphology, either [16, 17, 22, 23, 25, 27]. Strengths of the present study relate to the fairly large number of patients in both subgroups and the employment of thorough statistical analyses.

## Conclusions

In summary, semen quality of GCT patients was found to be significantly better prior to orchiectomy than in the first weeks after surgery. This result is consistent with four previous studies and the present study thus increases the weight of formal evidence for this finding. The superiority of preoperative ejaculate quality relates to all major semen parameters, specifically total sperm count but also ejaculate volume and sperm motility. The favourable quality of preoperative semen samples was equally evident in both seminomas and nonseminomas and also in various oncological subsettings. Obviously, testicles struck by cancer still are still a viable source for sperm retrieval. Although spontaneous recovery of semen quality in the later course is not improbable, GCT patients and care-givers of whom are best advised to consider sperm cryopreservation at the time before orchiectomy.

## Data Availability

The datasets used and analysed during the current study are available from the corresponding author on reasonable request.

## Abbreviations

AFP: Alpha fetoprotein
AKA: Asklepios Klinik Altona, Hamburg
bHCG: Beta human chorionic gonadotropin
BWKH: Bundeswehrkrankenhaus Hamburg
CI: Confidence interval
CS: Clinical stage
EV: Ejaculate volume
GCT: Germ cell tumour
ICSI: Intracytoplsamatic sperm injection
TESE: Testicular sperm extraction
TSC: Total sperm count
ULN: Upper limit of norm
WHO: World Health Organisation
